# A comprehensive analysis of novel disulfide bond introduction site into the constant domain of human Fab

**DOI:** 10.1038/s41598-021-92225-9

**Published:** 2021-06-21

**Authors:** Hitomi Nakamura, Moeka Yoshikawa, Naoko Oda-Ueda, Tadashi Ueda, Takatoshi Ohkuri

**Affiliations:** 1 Faculty of Pharmaceutical Sciences, Sojo University, 4-22-1 Ikeda, Nishi-ku, Kumamoto, 860-0082 Japan; 2 Graduate School of Pharmaceutical Sciences, Kyushu University, 3-1-1 Maidashi, Higashi-ku, Fukuoka, 812-8582 Japan

**Keywords:** Biochemistry, Molecular biology

## Abstract

Generally, intermolecular disulfide bond contribute to the conformational protein stability. To identify sites where intermolecular disulfide bond can be introduced into the Fab’s constant domain of the therapeutic IgG, Fab mutants were predicted using the MOE software, a molecular simulator, and expressed in *Pichia pastoris.* SDS-PAGE analysis of the prepared Fab mutants from *P. pastoris* indicated that among the nine analyzed Fab mutants, the F130C(H):Q124C(L), F174C(H):S176C(L), V177C(H):Q160C(L), F174C(H):S162C(L), F130C(H):S121C(L), and A145C(H):F116C(L) mutants mostly formed intermolecular disulfide bond. All these mutants showed increased thermal stability compared to that of Fab without intermolecular disulfide bond. In the other mutants, the intermolecular disulfide bond could not be completely formed, and the L132C(H):F118C(L) mutant showed only a slight decrease in binding activity and β-helix content, owing to the exertion of adverse intermolecular disulfide bond effects. Thus, our comprehensive analysis reveals that the introduction of intermolecular disulfide bond in the Fab’s constant domain is possible at various locations. These findings provide important insights for accomplishing human Fab stabilization.

## Introduction

Monoclonal antibodies are highly effective against intractable diseases, such as cancer and rheumatism^1–3^. While the IgG molecule has several advantages, it must be produced in mammalian cell lines, and such a production requires the applications of a superior technology, resulting in high costs associated with the production of the necessary therapeutic doses. Alternatively, antibody fragments, in particular the single-chain antibody fragment (scFv) and fragment antigen binding (Fab) can be efficiently expressed in microbial expression systems. Since Fab is more stable than scFv, it demonstrates increased suitability for clinical applications^4^, such as certolizumab pegol, which is used for the treatment of rheumatoid arthritis and Crohn’s disease^5^.

However, therapeutic proteins are at risk of degradation during the purification process or product storage, leading to protein aggregation or the appearance of antigenicity^6–10^. For instance, deamidation of the asparagine residues is one of the most frequently occurring modifications, which may cause changes in bioactivity, bioavailability, or immunogenicity^11–18^. Furthermore, it has been shown that the deamidation of Asn 30 in the light chain of a recombinant IgG reduces its binding potency to 70%^19^. While proteins usually maintain an equilibrium between the native and denatured states, the equilibrium considerably favors the native state under physiological conditions. If proteins are inherently unstable, the rate of transition from the native to the denatured state is relatively rapid, resulting in protein degradation^20–22^; therefore, the stabilization of therapeutic proteins is effective in suppressing protein degradation. Intermolecular disulfide bond generally contribute to the conformational protein stability. A previous study has shown that the introduction of intermolecular disulfide bond increased the conformational stability of dimer protein^23–26^. However, few attempts have been made to report the introduction of a novel intermolecular disulfide bond into the Fab molecule to increase protein stability (Supplementary Figures 1 and 2).

An expression system of a Fab of IgG1 developed as the therapeutic antibody adalimumab, an anti-human TNF-α, was constructed in the yeast strain *P. pastoris*. We targeted Fab's constant domain to promote the applicability of our findings to other human Fabs. Furthermore, we predicted the intermolecular disulfide bond mutation sites of adalimumab Fab using the Disulfide Scan modeling software, and further analyzed the first mutants, the Fab-H (V177C) and L chain (Q160C), to be successfully expressed in yeast^27^. The Fab mutant was confirmed to undergo thermal stabilization through the formation of an intermolecular disulfide bond. In this study, in order to identify the further sites that facilitated the introduction of intermolecular disulfide bond within the Fab’s constant domain and to collect information on its stabilization, other presumed mutants with engineered intermolecular disulfide bond were prepared using *P. pastoris*.

## Results

### Preparation of intermolecular SS mutants

**Figure 1:**
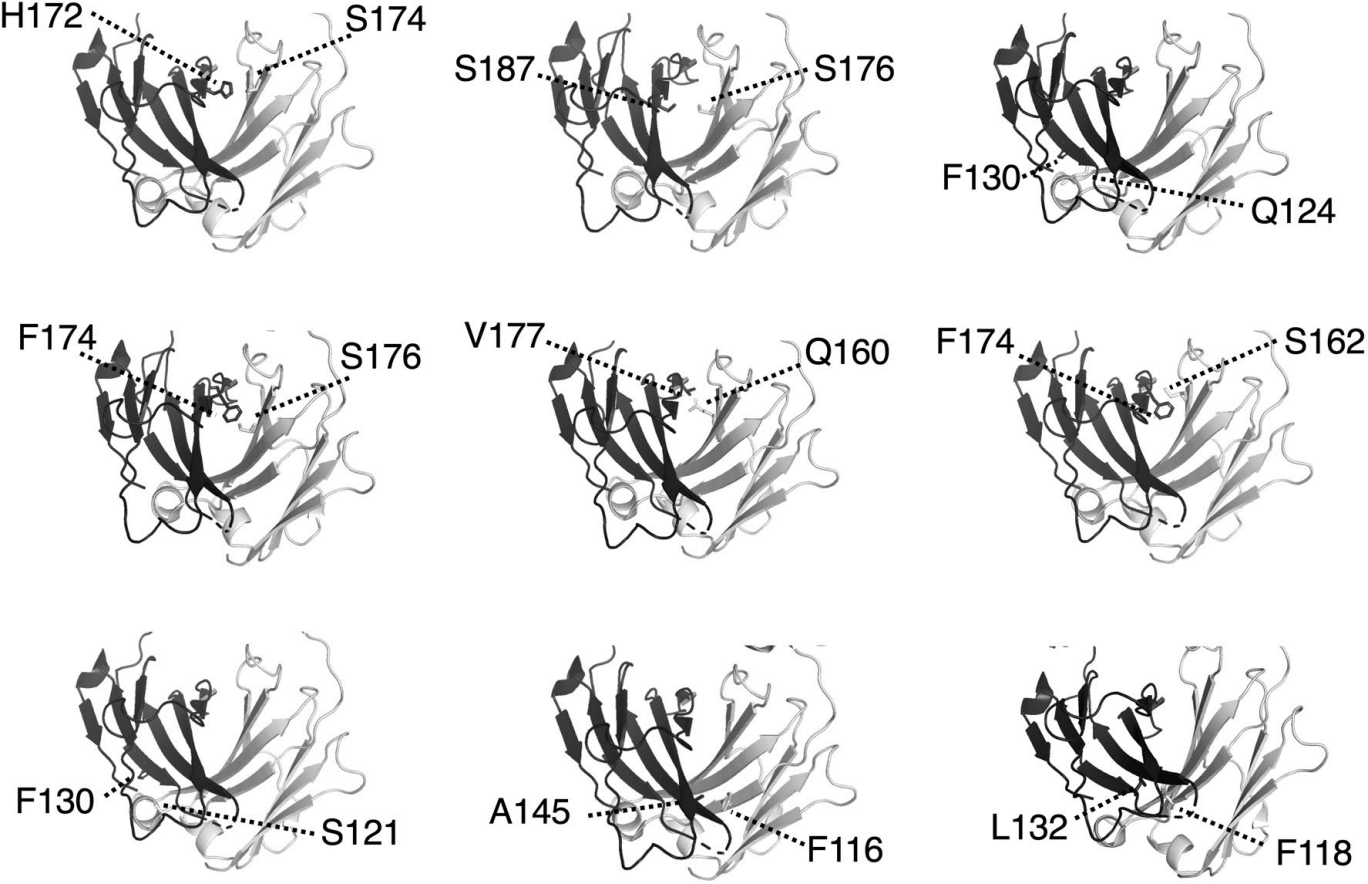
Mutation site of intermolecular SS mutants. Three-dimensional structure of adalimumab Fab (protein data bank number: 4NYL). The mutated amino acids are shown as sticks.

**Figure 2:**
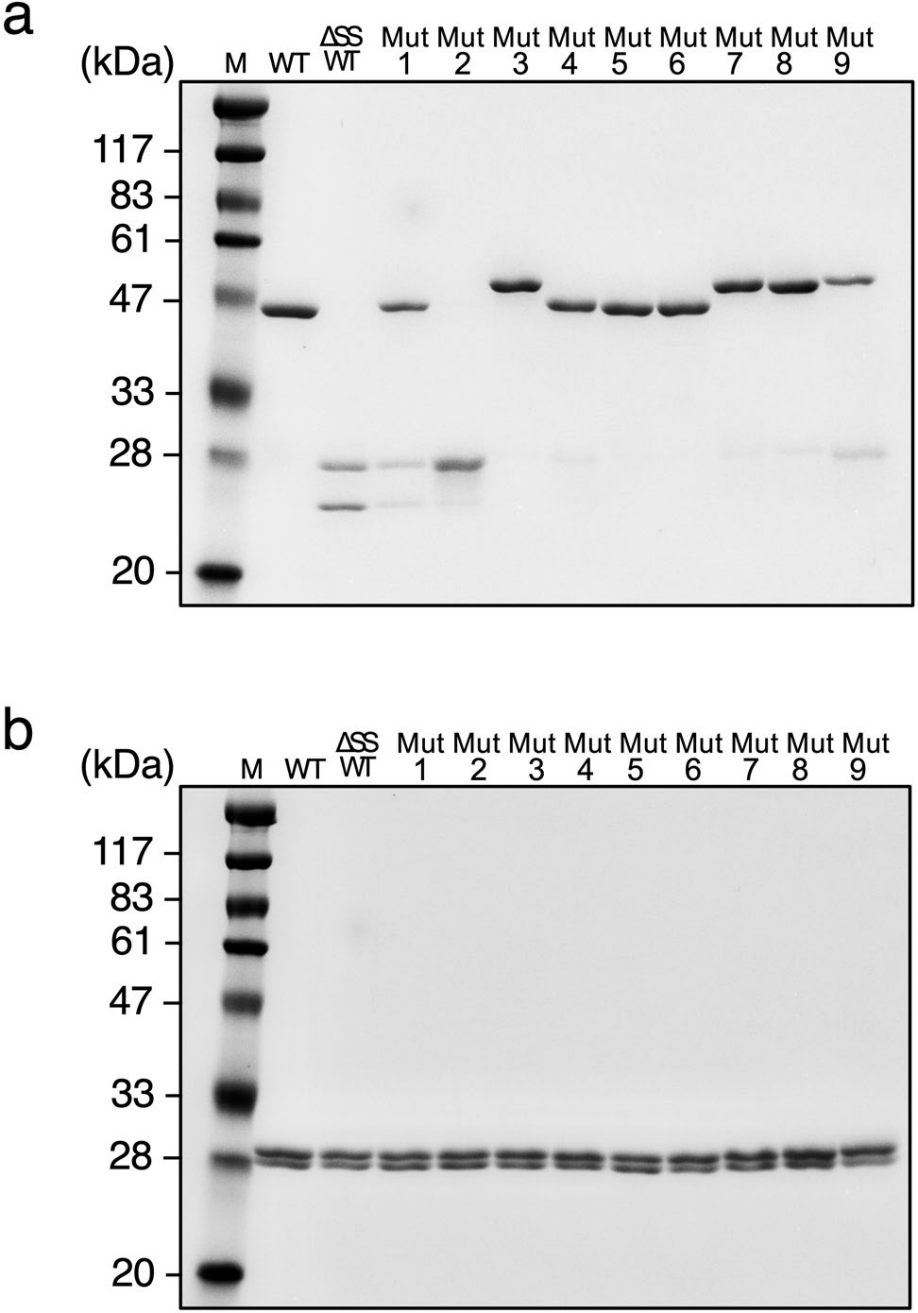
SDS-PAGE analysis of purified intermolecular SS mutants. The samples were analyzed by 12% SDS-PAGE under (**a**) non-reduced condition and (**b**) reduced condition. Lane M: protein markers. Lane 1: WT, Lane 2: ΔSSWT, Lane 3: Mut1, Lane 4: Mut2, Lane 5: Mut3, Lane 6: Mut4, Lane 7: Mut5, Lane 8: Mut6, Lane 9: Mut7, Lane 10: Mut8, Lane 11: Mut9. The gels were stained with SimplyBlue safe stain (Invitrogen) and were then imaged using iBright FL1000 (Thermo Fisher Scientific).

To introduce an intermolecular disulfide bond in the Fab’s constant domain, the prediction of disulfide bond formation by mutation was performed using the Disulfide Scan modeling software, and the structure of adalimumab Fab (protein data bank number: 4NYL) was used as a modeling template. The mutation sites of the prepared mutants (intermolecular SS mutants: Mut1–Mut9) and the distance between the β-carbon atoms present in the two residues are shown in Table 1. Data regarding the mutated amino acid residues were plotted on a three-dimensional structure of the adalimumab Fab as shown in Fig. 1. The wild-type Fab has one intermolecular disulfide bond at the C-terminal end. To facilitate the analysis of intermolecular disulfide bond formation, mutations were made to Fab in which the cysteine residues to form intact C-terminal intermolecular disulfide bond (C224(H):C214(L)) in human Fab were replaced with alanine (ΔSSWT); resulting that we can detect quickly whether or not an intermolecular disulfide bond in mutant Fabs form using SDS-PAGE of respective mutant treated in the absence or presence of reductant. The intermolecular SS mutants (Mut1–Mut9) were expressed in the yeast strain *P. pastoris* and purified as previous methods^28^. The final purified proteins were sufficiently obtained for all the mutants (Table 1). The SDS-PAGE of intermolecular SS mutants after the final purification step are shown in Fig. 2 (Supplementary Fig. 1). Under non-reducing conditions, the wild-type Fab (WT), which is connected at the C-terminal by the disulfide bond of the Fab-H (theoretical value: 23.8 kDa) and L chain (theoretical value: 23.4 kDa) showed a single protein band at approximately 47–50 kDa, while two bands were observed for ΔSSWT which were derived from the Fab-H and L chains. Furthermore, the intermolecular SS mutants were evaluated for disulfide bond formation based on the results obtained for WT and ΔSSWT (Fig. 2a). Mut1 exhibited both disulfide and non-disulfide formations, while Mut2 did not contain disulfide formations. Similarly, Mut9 exhibited a small number of disulfide forms. In contrast, Mut3, Mut4, Mut5, Mut6, Mut7, and Mut8 mostly formed intermolecular disulfide bond. Since the same results were obtained under reducing conditions, confirming that the protein bands of Fab-H and L chains were present in a 1:1 ratio for all intermolecular SS mutants (Fig. 2b).

**Table 1:** Intermolecular SS mutants.

Mutant	H chain site	L chain site	Cβ–Cβ (Å)	Introduce SS bond	Tm (°C)	Purified yield (mg/L)
Mut1	H172C	S174C	4.9	Incomplete	–	3.9
Mut2	S187C	S176C	4.8	Incomplete	–	4.6
Mut3	F130C	Q124C	4.8	Complete	74.0	6.5
Mut4	F174C	S176C	4.8	Complete	73.8	5.2
Mut5	V177C	Q160C	4.8	Complete	74.9	14.3
Mut6	F174C	S162C	4.2	Complete	72.7	9.2
Mut7	F130C	S121C	5	Complete	73.0	4.2
Mut8	A145C	F116C	4.2	Complete	73.3	9.7
Mut9	L132C	F118C	4.1	Incomplete	–	2.2

### Characterization of intermolecular SS mutants

**Figure 3:**
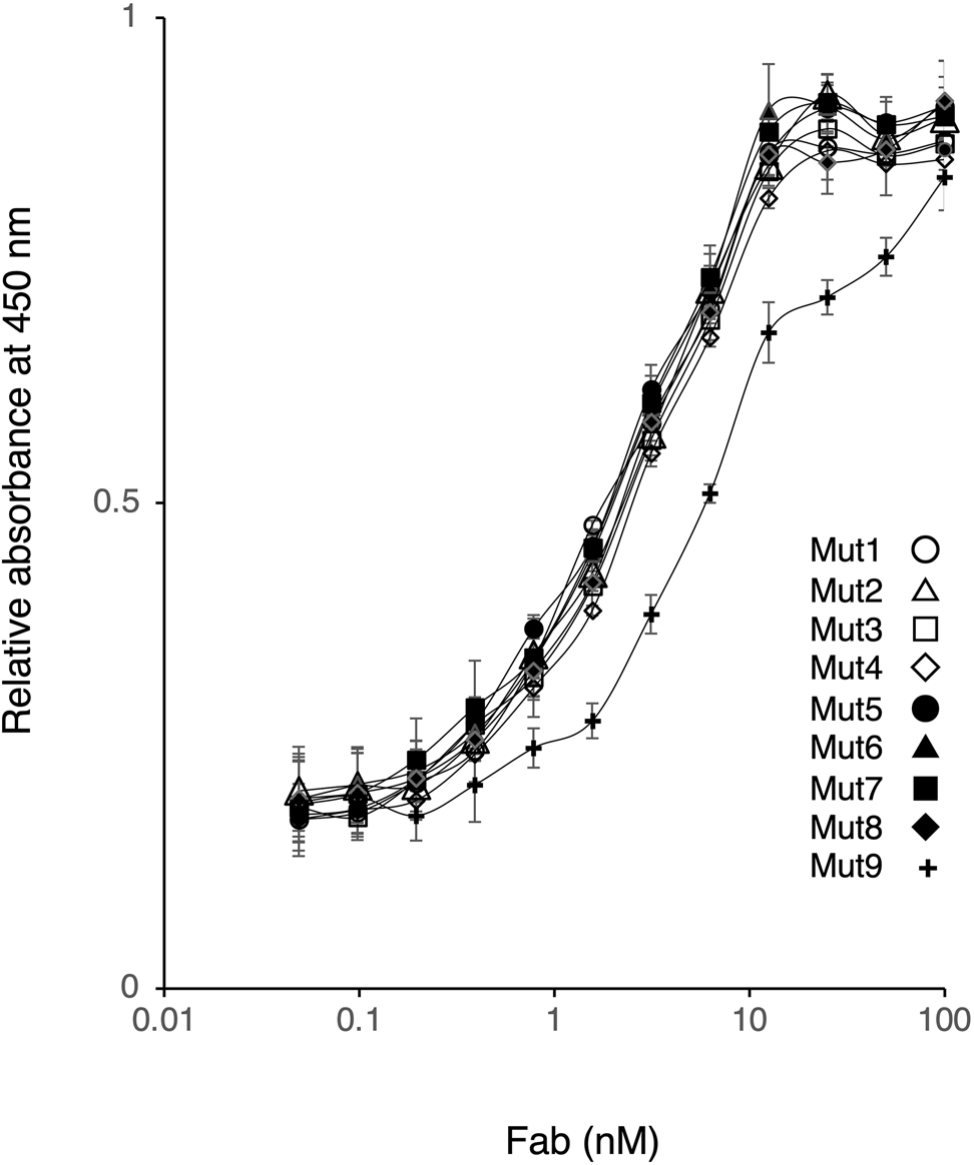
The antigen-binding activity of intermolecular SS mutants. The ELISA plate was coated with TNFα, followed by addition of intermolecular SS mutants at various concentrations. Anti-human Fab conjugated with horseradish peroxidase polyclonal antibody was used for evaluating the antigen-binding activity. The absorbance was measured at 450 nm using microplate spectrometer.

**Figure 4:**
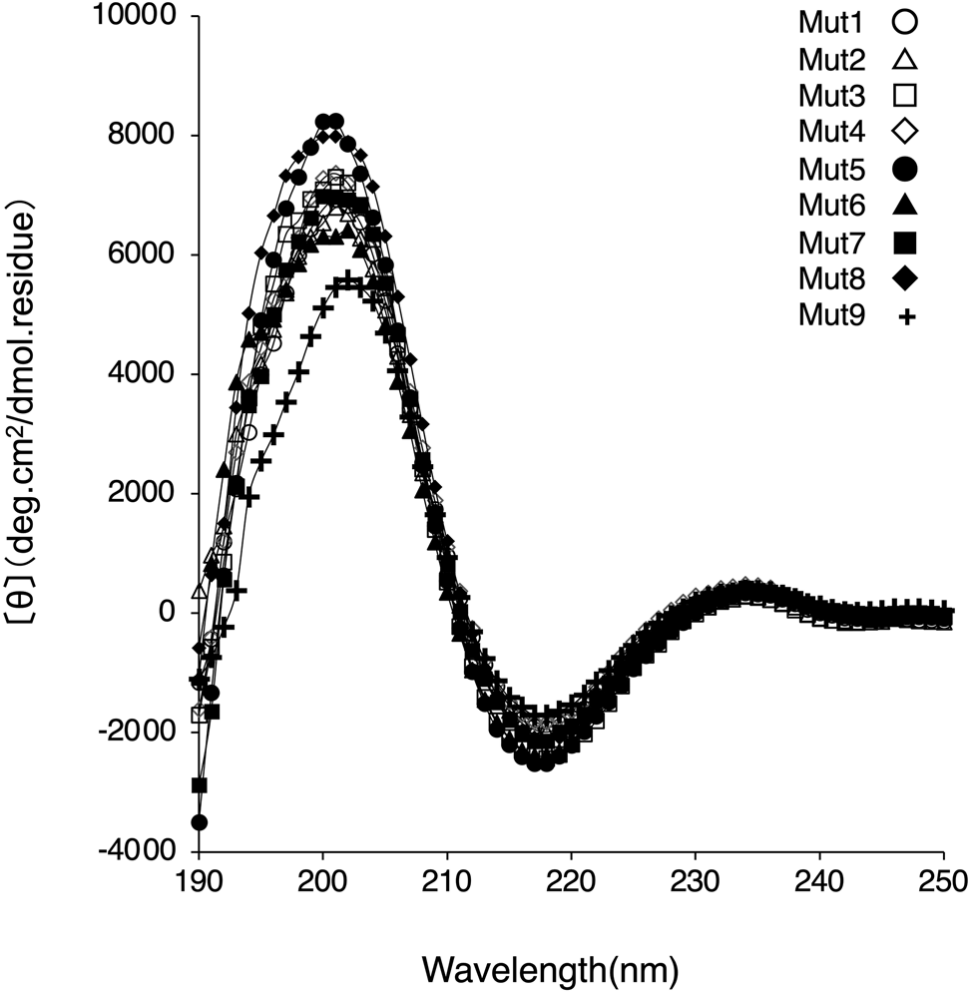
CD spectra of intermolecular SS mutants. The intermolecular SS mutants were dialyzed against 50 mM KH_2_PO_4_ buffer, pH 6.5, and prepared at a concentration of 0.2 mg/mL. Then, the CD spectra were collected on a spectropolarimeter.

The antigen-binding activity of intermolecular SS mutants was determined using ELISA (Fig. 3). While there was no difference in the 450 nm signal increase depending on the Fab concentration among all mutants, Mut9 showed a slight decrease in antigen-binding activity. The CD spectra of the intermolecular SS mutants were recorded in 50 mM KH_2_PO_4_ buffer, pH 6.5 (Fig. 4). The spectrum of Mut9 showed a slight decrease in β-helix content compared to the other mutants. Based on the results obtained, we concluded that the intermolecular disulfide bond introduction had almost no effect on the antigen-binding activity and structure of Fab, except for Mut9 wherein a slight effect was observed.

### Thermostability of intermolecular SS mutants

**Figure 5:**
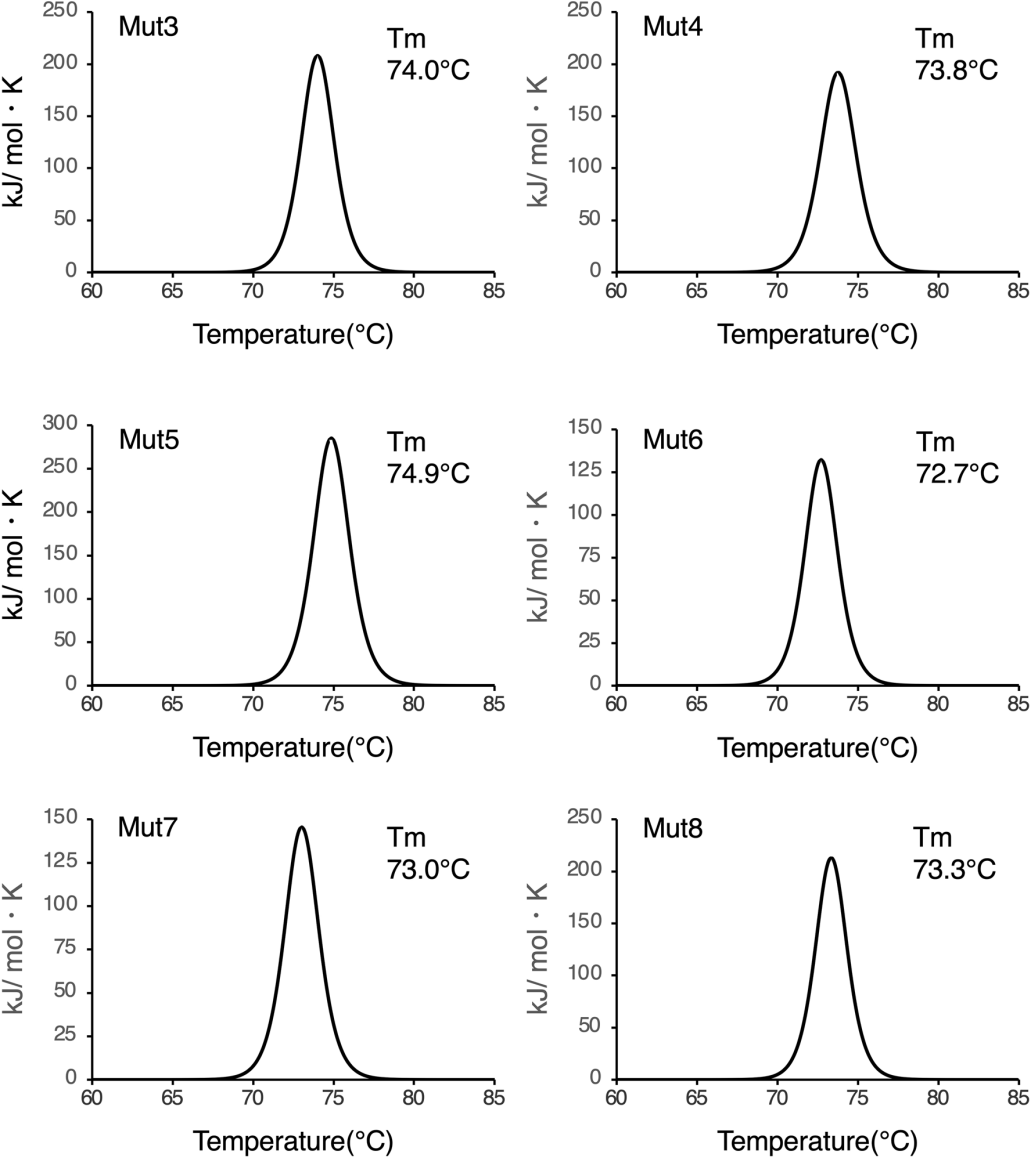
DSC measurements. Thermograms of Mut3, Mut4, Mut5, Mut6, Mut7, and Mut8 monitored from 25 and 90 °C at a scan rate of 60 °C/h. The protein solutions were prepared at concentration of 0.2 mg/mL in 50 mM KH_2_PO_4_ buffer, pH 6.5.

To investigate the contribution of the introduced intermolecular disulfide bond to thermostability, Mut3, Mut4, Mut5, Mut6, Mut7, and Mut8 were assessed using DSC (Fig. 5; Table 1). A previous study indicated that the melting temperature (Tm) values for ΔSSWT and WT were 69.9 °C and 74.9 °C, respectively^27^. The Tm values for intermolecular SS mutants, Mut3, Mut4, Mut6, Mut7, and Mut8 were higher than those of ΔSSWT, indicating that the introduction of intermolecular disulfide bond improved the Fab’s thermostability, as previously reported for Mut5.

### Refolding of WT and Mut1

**Figure 6:**
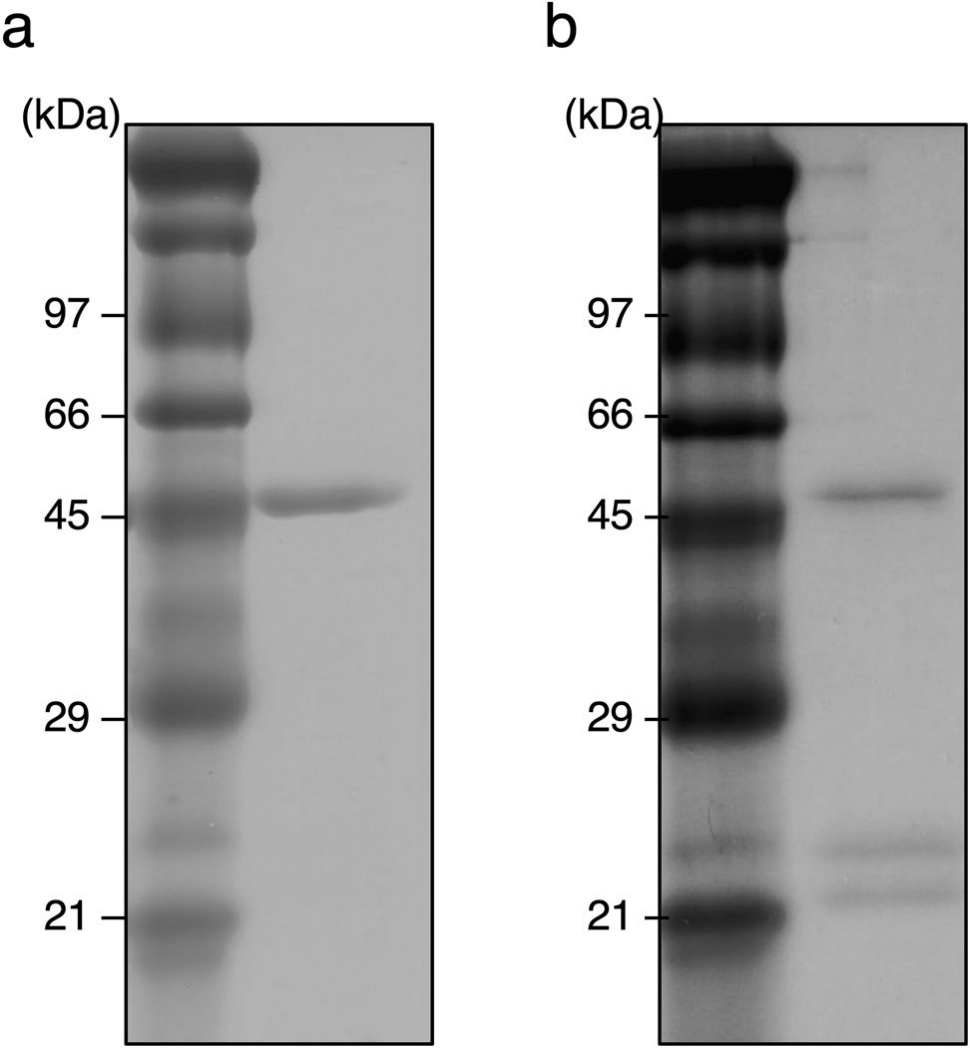
SDS-PAGE analysis of refolded Fab. The samples of (**a**) refolded WT and (**b**) refolded Mut1 analyzed by 12% SDS-PAGE under non-reduced condition. The gels were stained with SimplyBlue safe stain (Invitrogen) and were then imaged using iBright FL1000 (Thermo Fisher Scientific).

Refolding of WT and Mut1 from the reduced and denatured condition were performed using the stepwise dialysis method as previously described^29^ with slight modifications. The formation of intermolecular disulfide bond of refolded WT and Mut1 was analyzed using SDS-PAGE under non-reduced conditions (Fig. 6 and Supplementary Fig. 2). Refolded WT showed a single band at the position corresponding to the Fab that formed the intermolecular disulfide bond. Conversely, refolded Mut1 contained disulfide and non-disulfide forms similar to those expressed in *P. pastoris*.

## Discussion

In a previous study, it was observed that mutations in the adalimumab Fab-H chain (V177C) and L chain (Q160C) could successfully enable the introduction of an intermolecular disulfide bond in the constant domain (corresponding to Mut5)^27^. The screening of numerous yeast expression strains allowed Mut1, Mut2, Mut3, Mut4, Mut6, Mut7, Mut8, and Mut9 to be expressed and purified from *P. pastoris*. The mutation sites of the intermolecular SS mutants were located at the buried interface of the CH1 and CL domains (Fig. 1). In line with the phenotype observed in Mut5, the SDS-PAGE analysis of these intermolecular SS mutants revealed that Mut3, Mut4, Mut6, Mut7, and Mut8 formed mostly intermolecular disulfide bond (Fig. 2). Mut4, Mut5, and Mut6 show engineered disulfide bond located near the top of the constant domain, while those of Mut3, Mut7, and Mut8 are near the bottom (Fig. 1). The SDS-PAGE performed under non-reducing conditions revealed band differences between Mut3, Mut7, and Mut8 and WT or Mut4, Mut5, and Mut6, and such differences might be explained by the different disulfide bond positions. DSC analysis indicated that the Tm values of ΔSSWT obtained following thermal denaturation were 5 °C lower than those of WT, indicating that the intermolecular disulfide bond significantly contributed to the thermal stability of Fab^27^. Therefore, intermolecular SS mutants that formed mostly intermolecular disulfide bond were further analyzed using DSC (Fig. 5). Mut3, Mut4, Mut5, Mut6, Mut7, and Mut8 showed higher Tm values than ΔSSWT (Tm: 69.9 °C), thereby proving that the formation of intermolecular disulfide bond increased Fab’s thermal stability.

Tm values of Mut3, Mut4, Mut6, Mut7, and Mut8 were slightly lower than those of Mut5, which was equivalent to that of WT. Several hydrophobic amino acids are reportedly located at the interface of the CH1 and CL domains and play an important role in molecular interactions^30,31^. Dillon et al*.* reported findings on the designed Fab interface used for selective pairing of cognate H and L chains for the production of bispecific IgG. They identified that residue F128(H) (corresponding to F130 for adalimumab), F170(H) (corresponding to F174 for adalimumab), F116(L) and F118(L) showed the most considerable energy losses upon replacement by alanine using computational simulation^32^. It is suggested that these phenylalanine residues contributes sufficiently to conformational stability. When conducting molecular simulations based on the structure of adalimumab (PDB number: 4NYL), each phenylalanine residue was found to be involved in establishing subunit interaction at the CH1-CL interface as follows: F130(H) established interaction with Q124(L) via π–H bonding between the π-electron of aromatic ring and the Cβ proton of glutamine; F174(H) established interaction with S176(L) via π–H bonding between the π-electron of aromatic ring and the Cβ proton of serine; F116(L) established interaction with A145(H) and L135(L) using van der Waals forces. Since the mutations of Mut3, Mut4, Mut6, Mut7, and Mut8 included the substitution of phenylalanine as F130C(H), F174C(H), F116C(L), it is possible that the Fab’s thermostability was hindered by this substitution. Mut9, which exhibits insufficient intermolecular disulfide bond formation, is characterized by mutations of hydrophobic amino acids to cysteine in both the Fab-H and L chain, i.e., mutation of L132C(H):F118C(L). By conducting molecular simulations based on the structure of adalimumab (PDB number: 4NYL), it was found that L132(H) established interaction with F118(L) by π–H bonding between the Cβ proton of leucine and the π-electron of aromatic ring. Residue F118(L) also established interaction with V133(L) and L135(L) via hydrophobic interactions, indicating the mutation's significant effect of the mutation on the three-dimensional structure. Therefore, Mut9 seems to exert a slight effect not only on disulfide bond formation, but also on antigen binding and secondary structure (Figs. 3 and 4). Conversely, Mut1 and Mut2 showed insufficient intermolecular disulfide bond formation, although the highly hydrophobic amino acids were not substituted. To verify the possibility that the mutation site of cysteine was modified in yeast cells with a substance such as glutathione, Mut1 was refolded under reductive denaturation conditions. While the SDS-PAGE analysis of refolded Mut1 showed that it did not completely form intermolecular disulfide bond, the WT mostly formed intermolecular disulfide bond under identical conditions (Fig. 6). Similar results were obtained for Mut2 (data not shown). Thus, we hypothesized that the formation of intermolecular disulfide bond by Mut1 and Mut2 might not be possible because of the influence of the surrounding environment.

The methylotrophic yeast *P. pastoris* is a powerful tool for obtaining heterologous proteins. In this study, expression of Fab from the therapeutic antibody adalimumab in *P. pastoris* was possible. The expression systems of *P. pastoris* offer significant advantages over *E. coli* expression systems in the production of several heterologous eukaryotic proteins containing multiple disulfide bonds; this is because yeasts provide an intracellular folding environment similar to that observed in mammalian cells^33^. Therefore, these results may also be applied to expression in CHO cells used in the production of therapeutic antibodies. Although the constant domain of the Fab was targeted to promote the applicability of our findings to other human Fabs, it might also be important to verify whether similar results could be obtained using the V domain of other therapeutic antibodies.

The C-terminal intermolecular disulfide bond in the constant domain plays an important role in Fab’s stability^4,27^. Most therapeutic antibodies including adalimumab are IgG1 type and possess intermolecular disulfide bond between Cys233 (Kabat numbering) at the C-terminal of CH1 domain and Cys214 (Kabat numbering) at the C-terminal of CLκ domain. In contrast, IgG4 type molecules formed disulfide bond between Cys127 (Kabat numbering) of CH1 domain and Cys214 of the CLκ domain. Although these intermolecular disulfide bond are located at different positions in the amino acid sequence, they exist in spatially similar positions^34^. Peters et al. reported data for intermolecular disulfide bond arrangement in IgG4 that was achieved by mutation of C127S and via introduction of cysteine at the C-terminal of CH1^35^. They demonstrated that such engineered disulfide bond could help increase the thermal stability of the Fab domain of IgG4. However, the stability of the engineered IgG4 mutants was slightly less than that of the IgG1 wild-type. These results indicate that intermolecular disulfide bond at the C-terminal site are important for stability of the Fab domain, and that the stabilization by arrangement of intermolecular disulfide bond is a challenging approach. Vaks et al. also reported the engineering of the artificial disulfide bond between CH1 and CL of IgG1 for enabling the correct pairing of H and L chains of bispecific IgGs^36^. They designed seven mutant pairs and introduced each mutation into IgG molecules. Among the mutants, cysteine pair of Mut6 obtained from the present study was included; however, the effects of introducing disulfide bond formation on the thermal stability of Fab have not been investigated in detail.

In this study, we showed that intermolecular disulfide bond could be introduced at various positions in the human Fab’s constant domain and they contribute to its stability. The reason for deleting the C-terminal intermolecular disulfide bond of the Fab by substituting the two cysteines with alanine was to examine the effect of each introduced disulfide bond. It has been reported that a combination of disulfide bonds or the establishment of cross-linking via chemical modification could help achieve substantial overall improvement in the stability of a protein^37,38^. Therefore, it will be possible to efficiently achieve Fab stabilization by implementing a combination of multiple engineered disulfide bonds, based on the information of individual disulfide bonds obtained in this study. Moreover, the contribution of the newly introduced intermolecular disulfide bond (Mut1 through Mut9) towards the stability did not fully exceed that of the C-terminal disulfide bond, indicating the importance of the C-terminal disulfide bond for the thermal stability of the Fab. On the other hand, the rationale for application of approaches for site-specific modification of Fab based on the use of polyethylene glycol or cancer drugs is to ultimately target the intermolecular disulfide bond between the accessible cysteine residue at the C-terminal region. Previous studies have reported the utilization of the cysteine residues to enable the formation of intact C-terminal intermolecular disulfide bond in human Fab to establish links with polyethylene glycol, and the resultant Fab has been demonstrated to possess superior pharmacokinetics in mice^39,40^. Therefore, it was also important that introduction of an engineered intermolecular disulfide bond into the constant domain of the Fab was employed to mitigate destabilization due to the reduction of its intermolecular disulfide bond. In conclusion, the present study conducted on the introduction of disulfide bonds in human Fab is likely to be useful in the development of novel Fab therapeutics.

## Methods

### Design of intermolecular disulfide bond

A pair of mutations capable of forming disulfide bond in the constant domain between Fab-H chain and L chain were identified using the “Disulfide Scan” modeling software in the Molecular Operating Environment platform (MOE; MOLSIS Inc., Japan). Two potential residues are considered to be able to form a disulfide bond if the distance between the β-carbon are within 5 Å. The structure of adalimumab Fab (protein data bank number: 4NYL) was used as a modeling template.

### Construction of adalimumab Fab expression plasmid

Each expression cassette of the Fab-H chain and L chain genes was both inserted into the expression vector pPICZαA (Invitrogen, USA) and constructed as a co-expression plasmid according to previous study^27^. Site-directed mutagenesis for Fab gene were performed by PCR using mutagenic primer^41^. Each Fab-H and L chain gene was designed to attach an extra serine residue at the N-terminus for efficient processing of α-factor signal peptide^28^.

### Screening of high-level expression strains of intermolecular SS mutants

Yeast *Pichia pastoris* (X-33) was transformed by electroporation using a linearized co-expression plasmid by digestion with *Pme*I according manufacture protocol (Invitrogen, USA). The transformant cells were plated on YPDS medium containing 100 µg/mL of zeocin. The YPDS plate incubated at 30 °C for 3 days and a number of the larger colonies that grew on the plate were selected. The selected colonies were each inoculated in 2 mL of BMGY medium at 30 °C for 3 days with shaking. After growth culture was centrifuged at 3000×*g* for 7 min, the pellet was resuspended in 2 mL of BMMY medium and incubated at 30 °C with shaking. To induce expression of Fab mutants, methanol was added to the BMMY medium at final concentration of 1% every 24 h. After 4 days, the each culture supernatant was harvested by centrifugation at 8000×*g* for 7 min. Using the culture supernatant, the strain having the highest binding activity to TNFα by ELISA was selected as the highly expression strain of adalimumab Fab.

### Large scale expression and purification of intermolecular SS mutants

The selected expression strain were grown in 400 mL of BMGY medium and intermolecular SS mutants were expressed in 400 mL BMMY medium according to previous study^27^. The expressed Fab was purified by a slight modification of the previous methods^27^. Namely, the culture supernatant was brought to 60% ammonium sulfate saturation and removed the residual medium by centrifugation at 10,000×*g* for 15 min at 4 °C. The precipitation of protein was resuspended in 50 mM CH_3_COOH-CH_3_COONa buffer, pH 4. The solution after dialysis against same buffer was then bound to a SP Toyopearl 650 M column (2.2 cm × 12 cm; Tosoh, Japan) equilibrated with 50 mM CH_3_COOH–CH_3_COONa buffer, pH 4.0, and eluted with 1.0 M NaCl in the same buffer. The pooled protein was dialyzed against 50 mM CH_3_COOH–CH_3_COONa buffer, pH 5.0 and was loaded onto a Blue Sepharose 6 Fast Flow column (1.5 cm × 6 cm; GE Healthcare, USA) and equilibrated with 50 mM CH_3_COOH–CH_3_COONa buffer, pH 5.0. The protein was eluted with 0.4 M NaCl in the same buffer. The eluted fraction containing Fab was dialyzed against 50 mM CH_3_COOH–CH_3_COONa buffer, pH 5.0 and further purified by cation exchange chromatography using a resource S column (column volume was 1 mL; GE Healthcare, USA) with AKTA purifier system. The column was equilibrated with 50 mM CH_3_COOH–CH_3_COONa buffer, pH 5.0, and eluted with a 30 mL linear gradient of NaCl from 0–0.4 M in the same buffer.

### Antigen-binding activities of intermolecular SS mutants

The antigen-binding activities of intermolecular SS mutants for human TNFα were determined by ELISA as described previously^27^. The recombinant human TNFα was prepared using *Escherichia coli* expression systems^27^. The plate (F96 Nunc-immunoplate; Thermo Scientific, USA) was coated with TNFα (0.2 µM) in 50 mM NaHCO_3_ buffer, pH 9.6, at 4 °C overnight. The wells on plate were washed with 20 mM Tris–HCl, pH 7.6, containing 0.14 M NaCl and 0.05% Tween 20 (washing buffer) and then blocked for 60 min at RT with washing buffer containing 2% skim milk (blocking buffer). After the washing steps, the solutions containing intermolecular SS mutants were added (50 µL/well) and incubated for 60 min at RT. After washing step, the incubated with anti-human Fab conjugated with horseradish peroxidase polyclonal antibody (Abliance, France) for 60 min at RT. After washing step, substrate solution for peroxidase (ELISA POD Substrate TMB Solution Easy; Nacalai Tesque, Japan) was added (100 µL/well) and incubated at RT. The reaction was terminated by the addition of 1 M H_2_SO_4_ (100 µL/well). The absorbance was measured at 450 nm using microplate spectrometer (Microplate Photometer Multiskan FC; Thermo Scientific, USA).

### CD spectra of intermolecular SS mutants

The intermolecular SS mutants were prepared at a concentration of 0.2 mg/mL in 50 mM KH_2_PO_4_ buffer, pH 6.5 at room temperature. Then, the CD spectra were collected on a J720-spectropolarimeter (Jasco, Japan).

### Differential scanning calorimetry (DSC) measurements

DSC measurements were performed using a Nano DSC (TA-instrument, USA) as described previously^27^. Thermograms of intermolecular SS mutants were monitored from 25 and 90 °C at a scan rate of 60 °C/h. The protein solutions were prepared at concentration of 0.2 mg/mL in 50 mM KH_2_PO_4_ buffer, pH 6.5. The data was analyzed to obtain the temperature (*T*_m_) using Nanoanalyze software (TA-instrument, USA).

### Refolding of the wild-type and Fab mutants

Refolding of WT and Mut1 were performed using the stepwise dialysis method as previously described^29^ with slight modifications. Briefly, the lyophilized WT and Mut1 were dissolved in 0.1 M Tris–HCl buffer, 1 mM EDTA, pH 8.0 containing 8 M urea and reduced with cysteamine (final concentration of 30 mM) at 40 °C for 90 min. After denaturation and reduction, cystamine was added to the reduced solution at final concentration of 10 mM (redox solution) and incubated at 40 °C for 20 min. The redox solution was dialyzed against 0.1 M Tris–HCl buffer, 1 mM EDTA, 0.3 mM cysteamine, 0.1 mM cystamine, pH 8.0 (dialysis buffer) containing 4 M urea at 4 °C for 4 h. Then, the stepwise dialysis was carried out for dialysis buffer containing 2 M, 1 M, and 0 M urea every 12 h. After final stepwise dialysis, further dialysis was performed against 0.1 M Tris–HCl buffer, 1 mM EDTA, pH 8.0 in the absence of cysteamine and cystamine.

## Supplementary Information

Supplementary Figures.

## References

[1] Plückthun A, Skerra A (1989). Expression of functional antibody Fv and Fab fragments in *Escherichia**coli*. Methods Enzymol..

[2] King DJ (1992). Expression, purification and characterization of a mouse–human chimeric antibody and chimeric Fab' fragment. Biochem. J..

[3] Sun C, Wirsching P, Janda KD (2003). Enabling ScFvs as multi-drug carriers: A dendritic approach. Bioorg. Med. Chem..

[4] Röthlisberger D, Honegger A, Plückthun A (2005). Domain interactions in the Fab fragment: A comparative evaluation of the single-chain Fv and Fab format engineered with variable domains of different stability. J. Mol. Biol..

[5] Goel N, Stephens S (2010). Certolizumab pegol. MAbs.

[6] Cromwell ME, Hilario E, Jacobson F (2006). Protein aggregation and bioprocessing. AAPS. J..

[7] Shire SJ (2009). Formulation and manufacturability of biologics. Curr. Opin. Biotechnol..

[8] Vázquez-Rey M, Lang DA (2011). Aggregates in monoclonal antibody manufacturing processes. Biotechnol. Bioeng..

[9] Joshi V, Shivach T, Kumar V, Yadav N, Rathore A (2014). Avoiding antibody aggregation during processing: Establishing hold times. Biotechnol. J..

[10] Torisu T, Maruno T, Hamaji Y, Ohkubo T, Uchiyama S (2017). Synergistic effect of cavitation and agitation on protein aggregation. J. Pharm. Sci..

[11] Geiger T, Clarke S (1987). Deamidation, isomerization, and racemization at asparaginyl and aspartyl residues in peptides. Succinimide-linked reactions that contribute to protein degradation. J. Biol. Chem..

[12] Brange J, Langkjaer L, Havelund S, Vølund A (1992). Chemical stability of insulin. 1. Hydrolytic degradation during storage of pharmaceutical preparations. Pharm. Res..

[13] Tomizawa H, Yamada H, Imoto T (1994). The mechanism of irreversible inactivation of lysozyme at pH 4 and 100 degrees C. Biochemistry.

[14] Kosky AA, Razzaq UO, Treuheit MJ, Brems DN (1999). The effects of alpha-helix on the stability of Asn residues: Deamidation rates in peptides of varying helicity. Protein Sci..

[15] Fujii N, Matsumoto S, Hiroki K, Takemoto L (2001). Inversion and isomerization of Asp-58 residue in human alphaA-crystallin from normal aged lenses and cataractous lenses. Biochim. Biophys. Acta.

[16] Robinson NE, Robinson AB (2001). Deamidation of human proteins. Proc. Natl. Acad. Sci. U.S.A..

[17] Liu H, Gaza-Bulseco G, Faldu D, Chumsae C, Sun J (2008). Heterogeneity of monoclonal antibodies. J. Pharm. Sci..

[18] Luo Q (2011). Chemical modifications in therapeutic protein aggregates generated under different stress conditions. J. Biol. Chem..

[19] Harris RJ (2001). Identification of multiple sources of charge heterogeneity in a recombinant antibody. J. Chromatogr. B Biomed. Sci. Appl..

[20] Careri G, Fasella P, Gratton E (1975). Statistical time events in enzymes: A physical assessment. CRC Crit. Rev. Biochem..

[21] Cooper A (1984). Protein fluctuations and the thermodynamic uncertainty principle. Prog. Biophys. Mol. Biol..

[22] Závodszky P, Kardos J, SvingorPetsko GA (1998). Adjustment of conformational flexibility is a key event in the thermal adaptation of proteins. Proc. Natl. Acad. Sci. U.S.A..

[23] Gokhale RS, Agarwalla S, Francis VS, Santi DV, Balaram P (1994). Thermal stabilization of thymidylate synthase by engineering two disulfide bridges across the dimer interface. J. Mol. Biol..

[24] Velanker SS (1999). Disulfide engineering at the dimer interface of *Lactobacillus**casei* thymidylate synthase: Crystal structure of the T155C/E188C/C244T mutant. Protein Sci..

[25] Bunting KA, Cooper JB, Tickle IJ, Young DB (2002). Engineering of an intersubunit disulfide bridge in the iron-superoxide dismutase of *Mycobacterium**tuberculosis*. Arch. Biochem. Biophys..

[26] Ohkuri T (2010). A protein's conformational stability is an immunologically dominant factor: Evidence that free-energy barriers for protein unfolding limit the immunogenicity of foreign proteins. J. Immunol..

[27] Nakamura H, Oda-Ueda N, Ueda T, Ohkuri T (2018). A novel engineered interchain disulfide bond in the constant region enhances the thermostability of adalimumab Fab. Biochem. Biophys. Res. Commun..

[28] Ohkuri T, Murase E, Sun SL, Sugitani J, Ueda T (2013). Characterization of deamidation at Asn138 in L-chain of recombinant humanized Fab expressed from *Pichia**pastoris*. J. Biochem..

[29] Fujii T, Ohkuri T, Onodera R, Ueda T (2007). Stable supply of large amounts of human Fab from the inclusion bodies in *E. coli*. J. Biochem..

[30] Carredano E (2004). A novel and conserved pocket of human kappa-Fab fragments: Design, synthesis, and verification of directed affinity ligands. Protein Sci..

[31] Teerinen T, Valjakka J, Rouvinen J, Takkinen K (2006). Structure-based stability engineering of the mouse IgG1 Fab fragment by modifying constant domains. J. Mol. Biol..

[32] Dillon M, Yin Y, Zhou J, McCarty L, Ellerman D, Slaga D, Junttila TT, Han G, Sandoval W, Ovacik MA, Lin K, Hu Z, Shen A, Corn JE, Spiess C, Carter PJ (2017). Efficient production of bispecific IgG of different isotypes and species of origin in single mammalian cells. MAbs.

[33] Daly R, Hearn MT (2005). Expression of heterologous proteins in *Pichia**pastoris*: A useful experimental tool in protein engineering and production. J. Mol. Recognit..

[34] Saphire EO, Stanfield RL, Crispin MD, Parren PW, Rudd PM, Dwek RA, Burton DR, Wilson IA (2002). Contrasting IgG structures reveal extreme asymmetry and flexibility. J. Mol. Biol..

[35] Peters SJ, Smales CM, Henry AJ, Stephens PE, West S, Humphreys DP (2012). Engineering an improved IgG4 molecule with reduced disulfide bond heterogeneity and increased Fab domain thermal stability. J. Biol. Chem..

[36] Vaks L, Litvak-Greenfeld D, Dror S, Shefet-Carasso L, Matatov G, Nahary L, Shapira S, Hakim R, Alroy I, Benhar I (2018). Design principles for bispecific IgGs, opportunities and pitfalls of artificial disulfide bonds. Antibodies (Basel).

[37] Matsumura M, Signor G, Matthews BW (1989). Substantial increase of protein stability by multiple disulphide bonds. Nature.

[38] Ueda T, Masumoto K, Ishibashi R, So T, Imoto T (2000). Remarkable thermal stability of doubly intramolecularly cross-linked hen lysozyme. Protein Eng..

[39] Khalili H, Godwin A, Choi JW, Lever R, Brocchini S (2012). Comparative binding of disulfide-bridged PEG-Fabs. Bioconjug. Chem..

[40] Nakamura H, Anraku M, Oda-Ueda N, Ueda T, Ohkuri T (2020). C-terminal cysteine PEGylation of adalimumab Fab with an engineered interchain SS bond. Biol. Pharm. Bull..

[41] Mine S, Ueda T, Hashimoto Y, Tanaka Y, Imoto T (1999). High-level expression of uniformly 15N-labeled hen lysozyme in *Pichia**pastoris* and identification of the site in hen lysozyme where phosphate ion binds using NMR measurements. FEBS Lett..

