# Supplementary material for: A comprehensive analysis of novel disulfide bond introduction site into the constant domain of human Fab
Source: Sci Rep. 2021 Jun 21;11:12937. doi: 10.1038/s41598-021-92225-9 (PMC8217492; doi:10.1038/s41598-021-92225-9)
Supplement: Supplementary file 1 — Supplementary Figures. [file 41598_2021_92225_MOESM1_ESM.pdf]

A comprehensive analysis of novel disulfide bond introduction site into the constant domain of human Fab

Hitomi Nakamura[1], Moeka Yoshikawa[1], Naoko Oda-Ueda[1], Tadashi Ueda[2], Takatoshi Ohkuri[1]*.

[1]Faculty of Pharmaceutical Sciences, Sojo University

[2]Graduate School of Pharmaceutical Sciences, Kyushu University

***Corresponding author:** Takatoshi Ohkuri

E-mail address: ohkuri@ph.sojo-u.ac.jp

Supplementary Figure

(a)

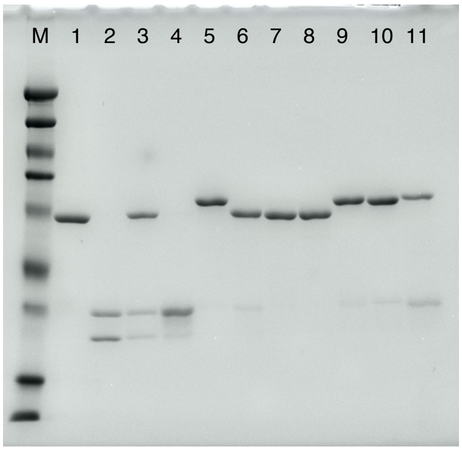

(b)

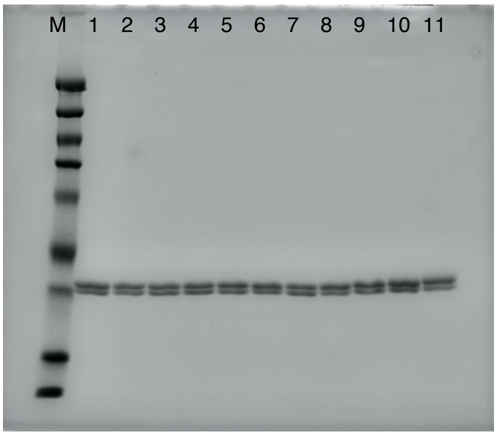

Supplementary Figure1. Original SDS-PAGE gels: Analysis of purified intermolecular SS mutants from Fig.1. The samples were analyzed by 12% SDS-PAGE under (a) non-reduced condition and (b) reduced condition. Lane M: protein markers. Lane 1: WT, Lane 2: ΔSSWT, Lane 3: Mut1, Lane 4: Mut2, Lane 5: Mut3, Lane 6: Mut4, Lane 7: Mut5, Lane 8: Mut6, Lane 9: Mut7, Lane 10: Mut8, Lane 11: Mut9. The gels were stained with SimplyBlue safe stain (Invitrogen) and were then imaged using iBright FL1000 (Thermo Fisher Scientific).

(a) (b)

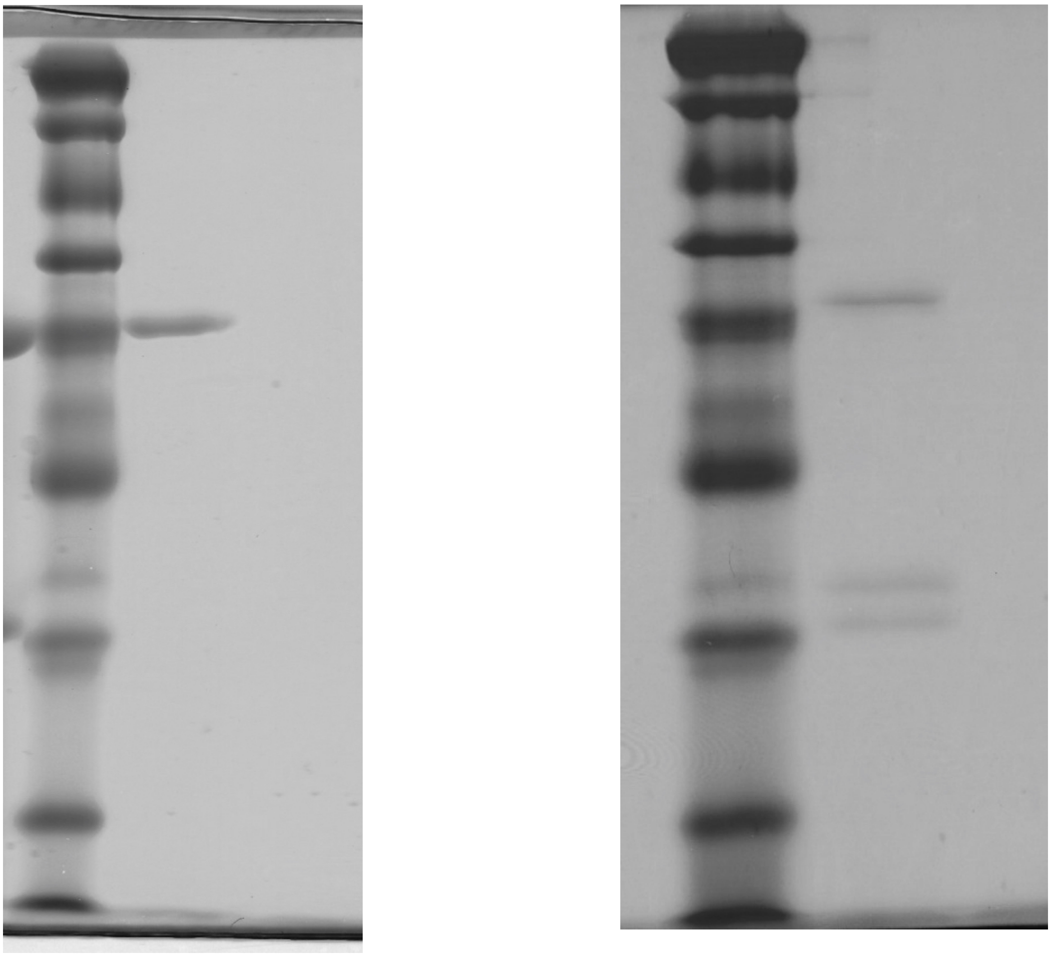

Supplementary Figure 2. Origenal SDS-PAGE gels: Analysis of refolded Fab from Fig.6. The samples of (a) refolded WT and (b) refolded Mut1 analyzed by 12% SDS-PAGE under non-reduced condition. The gels were stained with SimplyBlue safe stain (Invitrogen) and were then imaged using iBright FL1000 (Thermo Fisher Scientific).
